# Prognostic impact of microscopic residual disease after neoadjuvant chemotherapy in patients undergoing interval debulking surgery for advanced ovarian cancer

**DOI:** 10.1007/s00404-024-07775-w

**Published:** 2024-10-13

**Authors:** Violante Di Donato, Giuseppe Caruso, Tullio Golia D’Augè, Giorgia Perniola, Innocenza Palaia, Federica Tomao, Ludovico Muzii, Angelina Pernazza, Carlo Della Rocca, Giorgio Bogani, Pierluigi Benedetti Panici, Andrea Giannini

**Affiliations:** 1 Department of Maternal and Child Health and Urological Sciences, University of Rome Sapienza, Policlinico Umberto I, Viale del Policlinico 155, 00161 Rome, Italy; 2 Department of Medical-Surgical Sciences and Biotechnologies, University of Rome Sapienza, Rome, Italy; 3 Gynecologic Oncology Unit, Fondazione IRCCS Istituto Nazionale Dei Tumori Di Milano, Milan, Italy; 4 Unit of Gynecology, Department of Surgical and Medical Sciences and Translational Medicine, Sant’Andrea Hospital, Sapienza University of Rome, Rome, Italy

**Keywords:** Ovarian cancer, Neoadjuvant chemotherapy, Residual tumor, Interval debulking surgery, Microscopic residual disease

## Abstract

**Purpose:**

To determine the prognostic impact of microscopic residual disease after neoadjuvant chemotherapy (NACT) in patients undergoing interval debulking surgery (IDS) for advanced epithelial ovarian cancer (AEOC).

**Methods:**

Patients affected by FIGO stage IIIC–IV ovarian cancer undergoing IDS between October 2010 and April 2016 were selected. Progression-free survival (PFS) and overall survival (OS) were estimated using the Kaplan–Meier analysis.

**Results:**

In total, 98 patients were identified. Four patients (4.1%) were considered inoperable. Overall, 67 patients (out of 94; 71.3%) had macroscopic disease, equating Chemotherapy Response Score (CRS) 1 and 2, 7 (7.4%) had microscopic residuals, equating CRS3, rare CRS2, while 20 (21.3%) had both microscopic and macroscopic disease. Median OS and PFS were, respectively, 44 and 14 months in patients with no macroscopic residual disease (RD = 0) compared to 25 and 6 months, in patients with RD > 0 (OS: *p* = 0.001; PFS: *p* = 0.002). The median PFS was 9 months compared to 14 months for patients with more or less than 3 areas of microscopic disease at final pathologic evaluation (*p* = 0.04). The serum Ca125 dosage after NACT was higher in patients with RD > 0 compared to those without residue (986.31 ± 2240.7 µg/mL vs 215.72 ± 349.5 µg/mL; *p* = 0.01).

**Conclusion:**

Even in the absence of macroscopic disease after NACT, the persistence of microscopic residuals predicts a poorer prognosis among AEOC patients undergoing IDS, with a trend towards worse PFS for patients with more than three affected areas. Removing all fibrotic residuals eventually hiding microscopic disease during IDS represents the key to improving the prognosis of these patients.

## What does this study add to the clinical work

**Table:** 

On one hand, removing potentially hidden cancer cells could enhance the outcomes for advanced epithelial ovarian cancer patients. On the other hand, it is crucial to integrate the potential benefit of removing occult neoplastic foci with a balanced approach to surgical aggressiveness to minimize morbidity. CA125 levels after neoadjuvant chemotherapy can serve as a proxy for residual microscopic disease.	

## Introduction

Primary debulking surgery (PDS) followed by adjuvant chemotherapy represents the standard of care for advanced epithelial ovarian cancer (AEOC) [1–3]. The maximal surgical effort is often needed to achieve no residual disease (RD), known as the most important prognostic factor [4–8]. Interval debulking surgery (IDS) after neoadjuvant chemotherapy (NACT) is generally considered for patients with unresectable disease at diagnosis or for frail patients unlikely to tolerate aggressive procedures [9–11].

Recently, several randomized clinical trials have demonstrated the non-inferiority in terms of progression-free survival (PFS) and overall survival (OS) of IDS compared to PDS for patients likely to achieve suboptimal resection [12–15]. As a result, the number of patients undergoing IDS has been increasing significantly over the past few years [16, 17]. However, it remains controversial whether IDS should be as conservative as possible or should aim at removing all fibrotic residuals potentially hiding microscopic tumor foci.

Despite the validity of the Chemotherapy Response Score (CRS) in grading the pathological response of ovarian cancer after NACT, some controversies are still debated [18]. CRS1 corresponds to minimal or absent tumor response; CRS2 represents tumor with intermediate response while CRS3 is the complete or near complete pathological response, with residual disease absent, focal or multifocal (up to 2 mm). One issue is that the CRS3 group includes tumors with a pathological complete response, as well as those with a single or even multiple residual tumor foci, which are clearly very different conditions. The present study aims to evaluate the prognostic impact in terms of PFS and OS of the resection of all microscopic residual diseases after NACT in ovarian cancer patients undergoing IDS.

## Materials and methods

From October 2010 to April 2016, patients with AEOC who underwent NACT due to unresectable disease at the Department of Gynecology and Obstetrics of Sapienza University of Rome were enrolled. All the patients included were of Caucasian ethnicity. The interval debulking surgeries were conducted by a multidisciplinary team of gynecologic oncologists, all highly trained in advanced ovarian cancer surgery. These surgeons worked together to perform complete cytoreduction, aiming for RD = 0 resection (no visible residual disease). Where specific procedures, such as hepatobiliary resection or colorectal surgery, were required, dedicated surgeons were brought in to collaborate with the core team. All data were retrospectively extracted from our prospectively collected ovarian cancer surgical database, the study was conducted in accordance with ethical guidelines and obtained approval from the institutional review boards.

The inclusion criteria were: (1) histologically confirmed primary ovarian, fallopian tube, or peritoneal carcinoma; (2) FIGO stage IIIC–IV upon either radiological or pathological assessment; (3) IDS after NACT. Women with non-epithelial or borderline ovarian cancer, concomitant non-gynecologic primary cancers, incomplete medical records, recurrent disease and pregnancy status were excluded. Demographic, oncological and surgical data for each patient were collected following the Strengthening the Reporting of Observational Studies in Epidemiology (STROBE) guidelines to reduce any risk of bias.

Neoadjuvant chemotherapy regimens administered were platinum and taxane-based. All patients were treated with at least 3 NACT cycles; additional cycles were considered only if the disease was still surgically unresectable. All patients underwent IDS after NACT except in case of disease progression, unresectable disease or poor performance status. The preoperative staging and assessment of resectability were obtained through imaging and/or laparoscopic evaluation [19]. The imaging techniques used during the preoperative evaluation included transvaginal ultrasound, positron emission tomography (PET), and computed tomography (CT).

The maximal surgical effort with cytoreductive intent was always pursued to remove all macroscopic and microscopic disease; in fact, microscopic disease was suspected and resected in case of carcinoma-like tissues with a non-unanimous appearance or fibrotic areas resulting from the response to NACT [20]. Surgical specimens were evaluated macroscopically and sampled according to current guidelines, and then a histological diagnosis comprehensive of CRS and TNM staging was made [18]. In the histopathological report, the samples were divided according to their origin area: (1) pelvis (uterus, ovaries, tubes, pelvic peritoneum, and parametrium); (2) middle abdomen (small intestine, colon, paracolic spaces, omentum, and mesentery); (3) upper abdomen (liver, spleen, diaphragm, adrenal gland, and gallbladder). The surgical complexity was evaluated according to the score system described by Aletti et al*.* [21]. The Accordion Severity Grading System was used to describe postoperative complications within 30 days [22].

The follow-up schedule was as follows: (1) clinical evaluation and Ca125 dosage every 3 months during the first year from IDS, every 6 months during the second year, and then annually; (2) PET/CT or CT scan every 6 months during the first 2 years and then annually until the fifth year. Primary outcomes evaluated were OS (the time from the first day of NACT to death or last follow-up) and PFS (the time from the day after IDS to progression, death or last follow-up).

The incidence of events was analyzed for statistical significance using the Fisher exact test. Odds ratio (OR) and 95% confidence intervals (CIs) were estimated for each comparison. Predictive factors of intraoperative and postoperative complications were evaluated using univariate and multivariate models. Multivariable models were performed for variables with a p value ≤ 0.10 based on univariate analysis. All the results refer to a two-sided p value; a p value < 0.05 was considered statistically significant, whereas a p value between 0.05 and 0.10 was considered adequate to determine a significant trend worth mentioning.

Overall survival and PFS were estimated with the Kaplan–Meier method and different factors were evaluated for their association with OS and PFS based on fitting univariate and multivariable Cox proportional hazards models. The statistical analysis was performed using the statistical software IBM SPSS version 25.0.

## Results

**Fig. 1:**
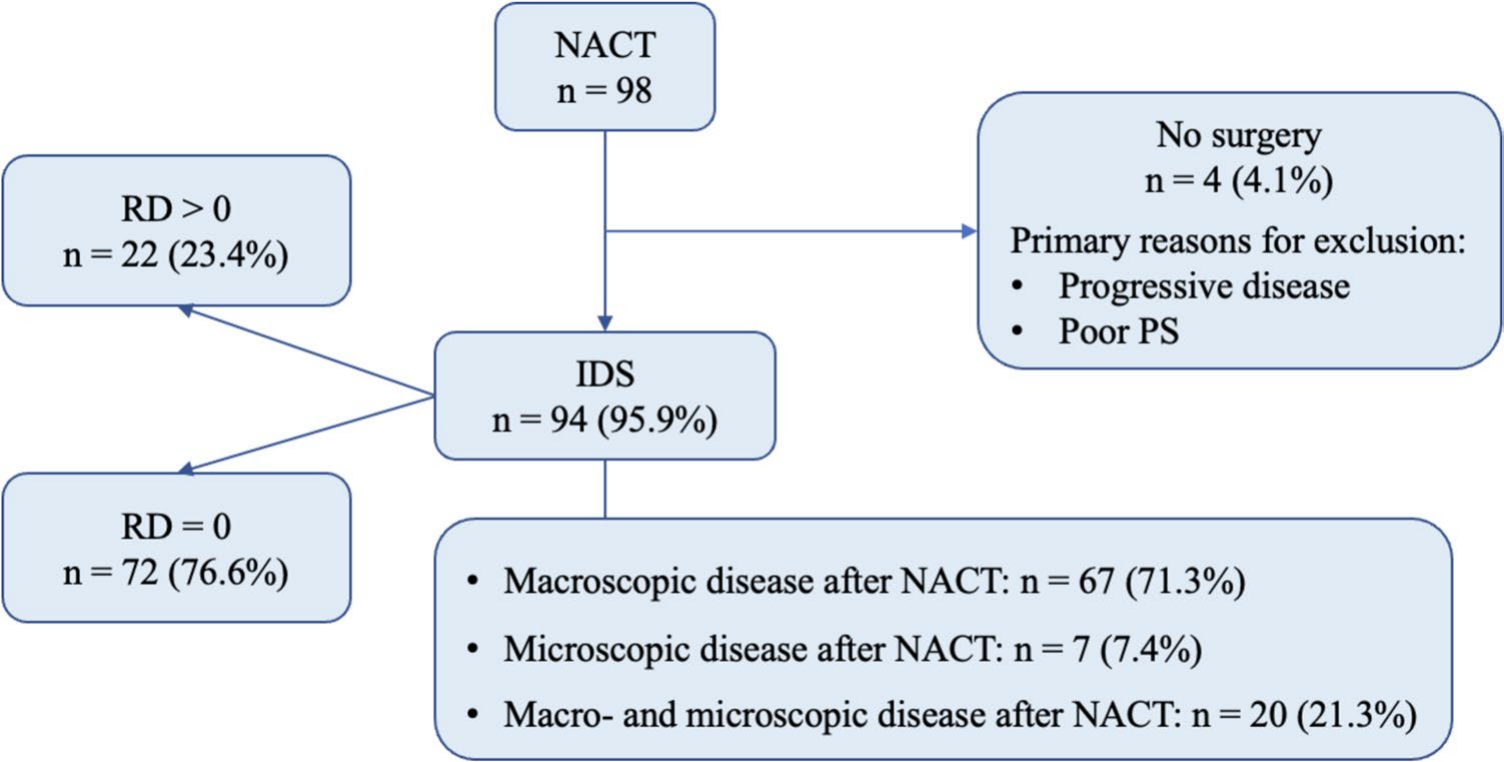
Flow chart of the study

A total of 98 patients with FIGO IIIC-IV stage ovarian cancer undergoing NACT were identified (Fig. 1). The characteristics of included patients are detailed in Table 1. The mean age was 59.1 ± 13.6 years and the mean BMI was 25.9 ± 4.6 kg/mq. The mean Ca125 value was 1631.6 ± 1935.57 µg/mL at diagnosis, 384.75 ± 1110.8 µg/mL after NACT, and 89.7 ± 164.9 µg/mL after IDS. Of the 98 patients, 64 (65.3%) underwent 3 NACT cycles, whereas 34 (34.7%) > 3 NACT cycles. Ninety-four (95.9%) patients underwent IDS with cytoreductive intent, whereas 4 (4.1%) patients were considered inoperable for progression of disease and/or poor performance status. Table 2 reports the surgical procedures performed and the surgical complexity index.

**Table 1:** Characteristics of patients

Variable	Patients (*n* = 98)
Age, mean ± SD (yrs)	59.1 ± 13.6
Lifestyle	
BMI, mean ± SD	25.9 ± 4.6
Smoker	18 (18.4%)
Ca125 (mean ± SD; µg/mL**)**	
Ca125 pre-treatment,	1631.6 ± 1935.57
Ca125 before surgery	384.7 ± 1110.8
Ca125 after surgery	89.8 ± 164.9
Grading	
G1	4 (4.1%)
G2	20 (20.4%)
G3	74 (75.5%)
FIGO stage	
IIIC	86 (87.7%)
IV	12 (12.3%)
Histological type	
Papillary serous	89 (90.8%)
Mucinous	4 (4,1%)
Endometrioid	3 (3.1%)
Clear cells	2 (2%)
Median n° Cycles NACT	3 (3–5)
Comorbidity (at least one)	
Hypertension	39 (39.8%)
Thyroid disease	23 (23.5%)
Diabetes	14 (14.3%)
Dyslipidemia	13 (13.3%)
Neurologic disease	9 (9.2%)
Tumors	8 (8.2%)
Gastrointestinal disease	7 (7.1%)
Infectious disease	6 (6.1%)
Cardiovascular disease	5 (5.1%)
Kidney disease	3 (3.1%)
Pelvic dysfunction	2 (2%)
Coagulation disorders	2 (2%)
AMI	0 (0%)
Respiratory disease	0 (0%)
mFI	
0	50 (51%)
1	31 (31,6%)
2	14 (14,3%)
3	1 (1%)
4	2 (2%)

*n* number, *SD* standard deviation, *BMI* Body mass Index, *FIGO* International Federation of Gynecology and Obstetrics, *AMI* Acute Myocardial Infarction, *NACT* neoadjuvant chemotherapy, *mFI* modified frailty index

**Table 2:** Surgical procedures on operated patients (n = 94 patients)

Procedures	Rates
Overall Rectal resection	31 (33%)
Anterior Rectal resection	5 (5.3%)
Segmental Rectal resection	26 (27.7%)
Omentectomy	93 (98.9%)
Appendicectomy	44 (46.8%)
Peritonectomy*	67 (71.3%)
Bowel resection	14 (14.9%)
Bulky node resection	9 (9.6%)
Ileal resection	7 (7.4%)
Gastric surgery	1 (1.1%)
Pancreatic surgery	8 (8.5%)
Splenic surgery	16 (17.1%)
Hepatic surgery	15 (15.9%)
Biliary surgery	18 (19.1%)
Diaphragmatic surgery	30 (31.9%)
Surgical complexity score	
Low (score ≤ 3)	19 (20.2%)
Intermediate (4–7)	47 (50%)
High (≥ 8)	28 (29.8%)
Complications	
Overall (G1–G5)	40 (42.5%)
Severe (G3–G5)	7 (7.4%)
90 days mortality	3 (3.2%)
Residual disease	
RD = 0	72 (76.6%)
RD > 0	22 (23.4%)

*RD* residual disease

^*^At least one of these areas: Bladder peritoneum, Douglas, paracolic gutter, mesentery

Briefly, 31 patients (33%) underwent rectal resection, 30 patients (31.9%) had diaphragmatic surgery and 33 patients (35.1%) hepatobiliary surgery. Nineteen patients (20.2%) received low-complexity surgery, whereas 47 (50%) and 28 (29.8%) patients underwent, respectively, intermediate and high-complexity surgery. Seventy-two patients (76.6%) achieved complete cytoreduction (RD = 0), whereas 22 (23.4%) had visible residual disease (RD > 0). Of the 22 patients not achieving complete resection, 10 patients (45.5%) had mesenteric involvement with superior mesenteric artery infiltration, 6 patients (27.3%) had small bowel diffuse carcinosis, in 2 patients (9.1%) the hepatic artery was infiltrated, 2 patients (9.1%) had common bile duct infiltration, one patient (4.5%) portal vein massive involvement and one (4.5%) multiple parenchymal hepatic metastasis. Overall, 40 patients (42.5%) had at least one postoperative complication, seven of whom (7/40; 17.5%) experienced severe complications (G3–G5). Three patients (3.2%) died within 90 days from surgery: one patient for pulmonary embolism and two for sepsis and multiorgan dysfunction syndrome (MODS). Of note, 78% (22/28) of patients undergoing high-complexity surgery developed postoperative complications compared with 43.9% (29/66) of patients who had a low (score ≤ 3) or intermediate (score 4–7) complexity surgery (*p* = 0.01). No patient had a complete pathological response after NACT at the histopathological assessment of the resected areas: 67 (71.3%) patients had macroscopic disease (CRS1-2), 7 (7.4%) subjects had microscopic residuals without macroscopic disease (CRS2-3), while 21.3% (20/94) had both microscopic and macroscopic disease. In particular, microscopic disease was found in the pelvis in 10.6% (10/94) of cases, in the middle abdomen in 9.6% (9/94), and in the upper abdomen in 2.1% (2/94).

Among the patients who achieved no visible residual tumor after interval debulking surgery (RD = 0), histopathologic evaluation of all resected peritoneal surfaces revealed at least one microscopic tumor (< 2 mm) in 18 patients (25%), which may include micro and macrometastases. Only microscopic disease without macrometastases was found in 7 patients (9.7%) (Table 3).

**Table 3:** Pathological finding

Location	Type of pathological finding			Missing data
	Macroscopic	Microscopic*	Absent	
Ovaries	76 (83.5%)	10 (11%)	5 (5.5%)	3 (3.2%)
Fallopian tubes	43 (67.2%)	1 (1.6%)	20 (31.2%)	30 (31.9%)
Uterus	33 (45.8%)	4 (5.5%)	35 (48.6%)	22 (23.4%)
Parametrium	32 (64%)	0 (0%)	18 (36%)	44 (46.8%)
Parametrium dx	33 (58.6%)	2 (3.6%)	21 (37.5%)	38 (40.4%)
Bladder peritoneum	34 (70.8%)	4 (8.3%)	10 (20.8%)	46 (48.9%)
Douglas	42 (70%)	3 (5%)	15 (25%)	34 (37.7%)
Left paracolic gutter	29 (69%)	3 (7.1%)	10 (23.8%)	52 (57.1%)
Right paracolic gutter	31 (64.5%)	4 (8.3%)	13 (27%)	39 (42.8%)
Omentum	70 (76.9%)	9 (9.9%)	12 (13.1%)	3 (5.1%)
Mesentery	29 (61.7%)	6 (12.8%)	12 (25.5%)	51 (52%)
Appendix	19 (44.2%)	3 (7%)	21 (48.8%)	51 (55.1%)
Gallbladder	2 (18.2%)	0 (0%)	9 (81.8%)	83 (88.8%)
Spleen	10 (62.5%)	0 (0%)	6 (37.5%)	78 (83.7%)
Diaphragm	15 (50%)	3 (10%)	12 (40%)	64 (69.4%)
Adrenal gland	1 (50%)	0 (0%)	1 (50%)	92 (98%)
Liver	4 (100%)	0 (0%)	0 (0%)	90 (95.9%)
Pelvis	81 (87.1%)	10 (10.6%)	2 (2.1%)	1 (1.1%)
Middle abdomen	77 (82.8%)	9 (9.6%)	7 (7.5%)	1 (1.1%)
Upper abdomen	23 (24.7%)	2 (2.1%)	68 (73.1%)	1 (1.1%)
Total	85 (91.4%)	7 (7.5%)	1 (1.1%)	1 (1.1%)

^*^Only in case of no macroscopic tumor in other areas

The median OS was 44 months for patients with RD = 0 compared to 25 months for those with RD > 0 (*p* = 0.001). The median progression-free survival (PFS) was 12 months: 14 months in the RD = 0 group and 6 months in the RD > 0 group (*p* = 0.002). The median PFS in the RD = 0 group for patients with more than 3 areas of microscopic disease at the final pathological evaluation was 9 months, compared to 14 months for those with fewer than 3 areas of microscopic disease (*p* = 0.04). The median OS was 44 months in the group with fewer than 3 areas of microscopic disease, compared to 35 months in those with more than 3 affected areas (*p* = 0.22). Of note, the mean Ca125 after NACT was significantly higher in patients with microscopic disease (986.31 ± 2240.7 µg/mL) compared to patients with no microscopic disease (215.72 ± 349.5 µg/mL) at final pathological evaluation (*p* = 0.01). Finally, no statistically significant difference in the occurrence of microscopic disease at pathological evaluation was found when comparing patients treated with up to 3 NACT cycles and those who received more than 3 cycles (20.7% vs 22%; *p* = 0.5).

## Discussion

In the present analysis, a microscopic residual disease without macroscopic disease at final pathological evaluation was recorded in 7.4% of patients after IDS, mainly involving the pelvis and the middle abdomen. The total amount of microscopic lesions demonstrated to have a significant impact on relapse rates, especially when the affected areas are 3 or more. The Ca125 level is a good proxy for predicting the presence of microscopic disease, yet the precise cutoff value remains unclear. This biomarker, often used in the monitoring of ovarian cancer, can indicate disease activity, but determining an exact threshold for the presence of microscopic disease involves complexities due to individual variations and the influence of other factors [23]. These considerations suggest a possible subclassification of CRS3, to identify tumors with better response to therapy and consequently significantly improve clinical outcomes.

Disposing of a marker able to predict the extension of microscopic residual disease after NACT could be the turning point for defining the surgical complexity required to obtain optimal cytoreduction. In our analysis, the Ca125 dosage after NACT was found to be a reliable predictive biomarker of the presence of areas with microscopical disease. These results are in agreement with those previously published in the literature [24].

Even if NACT is generally associated with a lower surgical complexity, a careful abdominal inspection is required in the course of IDS to discover and remove occult neoplastic microscopic disease [4]. Of note, the occurrence and the amount of microscopic residuals after NACT were not related to the number of chemotherapy cycles administered. Removing suspicious white areas could be an effective strategy to reduce the likelihood of leaving behind macroscopic diseases that are not visible to the naked eye. This surgical strategy could aim to target areas that may harbor occult disease, enhancing the thoroughness of the debulking procedure. By focusing on these suspicious areas, surgeons can potentially improve the chances of achieving a more complete cytoreduction, which is crucial for improving patient outcomes in diseases like ovarian cancer, where residual disease significantly impacts prognosis.

However, it is of paramount importance to balance the effects of surgery-related morbidity with the potential benefits of removing potential neoplastic foci that could lead to resistance [25–27]. This delicate balance requires careful consideration of the immediate risks associated with more extensive surgical interventions against the long-term benefits of potentially reducing the risk of chemoresistant disease recurrence. Decisions in this context are complex and must be tailored to the individual patient's condition, prognosis, and overall health, emphasizing the importance of a multidisciplinary approach in treatment planning [1, 2, 28].

Where surgery can easily remove superficial lesions (for example, peritoneal, mesenteric, or diaphragmatic surfaces) with minimal morbidity, it is always advisable to excise these potential lesions [29, 30]. However, the selection of these interventions necessitates a comprehensive evaluation of the patient's health status, potential impact on their quality of life and disease extension [31, 32].

If suspicious areas are located in regions where surgery could lead to a higher rate of surgical morbidity or require highly complex surgery, the risk–benefit ratio of extended surgical aggressiveness must be carefully evaluated. Decision-making in such scenarios requires a multidimensional approach, considering not only surgical feasibility but also tolerability in light of a woman's frailty and, importantly, the patient's preferences, to determine the most appropriate course of action [19, 32]. This perspective emphasizes the need for a patient-centered approach in treatment planning, particularly in vulnerable populations. In the present series, post-NACT fibrotic residuals scattered within delicate areas such as the stomach, liver, or intestinal loops were treated with neutral argon plasma energy to minimize surgical morbidity risk while attempting to reduce the chance of microscopic disease persistence.

Such precision in operative planning would be facilitated by a radiomics-based prediction model of histopathologic response to neoadjuvant chemotherapy or intraoperative molecular imaging, enabling a more targeted and less invasive approach to surgical oncology [33].

A recent phase III multicentric study explored the application of pafolacianine in conjunction with near-infrared imaging for the real-time detection of folate receptor-positive ovarian cancer. Remarkably, in approximately 40% of the patients, this innovative approach significantly identified additional cancerous tissues that were not initially marked for resection and remained undetected through traditional white light assessment and palpation. This finding was particularly relevant for patients undergoing interval debulking surgery, suggesting that pafolacianine could play a crucial role in enhancing the precision of surgical interventions [34].

This approach would represent a major step forward in personalized cancer care, focusing on maximizing therapeutic efficacy while minimizing unnecessary interventions and their associated risks.

The limitations of this study are related to its retrospective nature and the relatively small cohort of patients enrolled, which may have led to selection and information bias. The strengths of the current study include meticulous observation within a dedicated oncological gynecology center and the availability of data collected in a prospective database of histological outcomes of areas not identified as tumorous for many patients.

The present study highlights the significance of heightened vigilance towards microscopic disease, in patients undergoing Interval Debulking Surgery (IDS), even when a complete response to Neoadjuvant Chemotherapy appears to be indicated by radiological or laparoscopic assessments. This suggests that despite conventional indicators showing apparent treatment success, the persistence of microscopic disease, may pose a significant risk to patients, highlighting the need for more sophisticated diagnostic and therapeutic strategies to identify and address such disease remnants. Indeed, the prognosis is influenced not only by the presence of macroscopic residual disease but also by microscopic neoplastic residuals [18]. This underscores the importance of thorough disease management beyond visible lesions, as microscopic remnants can significantly impact patient outcomes even in the absence of detectable macroscopic disease. These considerations suggest a possible subclassification of CRS3, to identify patients with absence or single focus of disease and patients with multifocal residual tumor, the latter with a high risk of recurrence [35].

Theoretically, achieving the absence of residual disease during Interval Debulking Surgery (IDS) should be more straightforward and associated with fewer postoperative complications. However, the response to Neoadjuvant Chemotherapy (NACT) is often inadequately assessed, particularly in cases of partial response. It is relatively common to discover microscopic residual disease within fibrotic areas caused by chemotherapy, which can elude even the most meticulous surgical exploration [18]. This discrepancy highlights the challenge of accurately evaluating the effectiveness of NACT and the need for enhanced diagnostic techniques to detect microscopic disease, ensuring more comprehensive treatment approaches.

A significant proportion of patients exhibit microscopic disease that is either undetected by imaging or difficult to assess during laparoscopy. Proper sampling of resected specimens with no visible or minimal residual tumor is crucial, as microscopic disease could be misdiagnosed. For example, during omental assessment, which can be challenging, it is recommended to take at least 4–6 histological sections [18].

## Conclusions

In conclusion, our data suggest that residual disease should be intended as the sum of microscopic and macroscopic diseases. Removing occult microscopic neoplastic foci could represent the key to improving the prognosis of these patients. The customization of surgical treatment in women undergoing NACT must balance not only the possibility of removing potential chemoresistant micrometastases but also the risk of surgery-related complications.

## Data Availability

All data relevant to the study are included in the article. Data are available upon appropriate request.
